# Resveratrol induces autophagy-dependent apoptosis in HL-60 cells

**DOI:** 10.1186/s12885-018-4504-5

**Published:** 2018-05-22

**Authors:** Yingying Fan, Jen-Fu Chiu, Jing Liu, Yan Deng, Cheng Xu, Jun Zhang, Guanwu Li

**Affiliations:** 1 Open Laboratory for Tumor Molecular Biology/Department of Biochemistry/The Key Laboratory of Molecular Biology for High Cancer Incidence Coastal Chaoshan Area, Shantou University Medical College, Xinling Road 22, Shantou, China; 2 Cheung Kong Scholar Laboratory, Shantou University Medical College, Xinling Road 22, Shantou, China; 3 Respiratory Department, The first Affiliated Hospital of Shantou University Medical College, Changping Road 57, Shantou, China

**Keywords:** Resveratrol, Apoptosis, Autophagy, Cell death, PI3K-Akt, AMPK-mTOR, HL-60

## Abstract

**Background:**

All known mechanisms of apoptosis induced by resveratrol act through cell cycle arrest and changes in mitochondrial membrane potential. It is currently unknown whether resveratrol-induced apoptosis is associated with other physiological processes, such as autophagy.

**Methods:**

Apoptosis-related markers involved in the intrinsic and extrinsic apoptotic pathways, and autophagic markers were detected by using western blotting and immunofluorescence. Mitochondrial membrane potential was assayed by flow cytometry. Pharmaceutical or genetic inhibition of autophagy involved were carried by 3- methyladenine or knockdown of autophagy-related (Atg) genes by siRNA. Differences between two values were tested by Student’s unpaired t test.

**Results:**

We show that resveratrol-induced apoptosis occurs through both the intrinsic and extrinsic apoptotic pathways. Mitochondrial membrane potential and apoptosis-related markers, such as an increased Bax/Bcl-2 ratio, and cleaved forms of caspase-8 and caspase-3, arise following resveratrol addition. Moreover, we find that resveratrol increases both the levels of microtubule-associated protein 1 light chain 3-II and the number of autophagosomes, and further demonstrate that resveratrol-induced autophagy depends on the LKB1-AMPK-mTOR pathway. We next reveal that some apoptosis-related markers induced by resveratrol are further attenuated by the inhibition of autophagy with 3-methyladenine or knockdown of autophagy-related (Atg) genes by siRNA.

**Conclusions:**

These results suggest that resveratrol induced apoptotic cell death of HL-60 cells depends on the autophagy activated through both the LKB1-AMPK and PI3K/AKT-regulated mTOR signaling pathways.

**Electronic supplementary material:**

The online version of this article (10.1186/s12885-018-4504-5) contains supplementary material, which is available to authorized users.

## Background

Resveratrol (trans-3, 4′, 5-trihydroxystilbene; RSV) was originally identified as a naturally occurring anti-tumor molecule. RSV is a polyphenol phytoalexin produced by several plants including grapes, blueberries and other plants [1, 2]. It has been reported to have antioxidant and anti-tumorigenic activities [3, 4]. Reports also show that RSV not only has the ability to inhibit tumor initiation and promotion, but also arrest metastasis [5, 6], and induce apoptosis [7–9]. Our previsous studies have indicated that RSV can inhibit the proliferation of human promyelocytic leukemia HL-60 cells by apoptosis in vitro [10]. Although recent studies on RSV induced autophagy in HL-60 cells have also attracted much attention [11], the accurate mechanisms and the roles of cell autophagy in apoptosis induced by RSV and the crosstalk between autophagy and apoptosis in HL-60 cells has not yet been fully established.

Autophagy is a highly conservative cell physiological process in eukaryotic organisms and is involved in the circulating in the cell components [12, 13]. It is a passive process that plays an important role in biological events, such as changes in environmental conditions, cell reconstruction and lifespan determination [14, 15]. In contrast to autophagy, apoptosis is programmed cell-death process characterized by membrane bubble, DNA fragmentation and unique apoptotic bodies [16, 17]. Apoptosis requires gene activation, expression and regulation, and is neither a pathological condition nor a phenomenon of self-injury, but rather a better adaptation to the environment and a proactive mechanism for death [18]. Here we report that RSV enhances autophagic flux and apoptosis simultaneously in a dose- and time-dependent manner in HL-60 cells. Furthermore, we demonstrate that RSV-induced HL-60 cell death involves autophagy-dependent apoptotic cell death via both the LKB1-AMPK and PI3K/AKT-regulated mTOR signaling pathways.

## Methods

### Chemicals and antibodies

A caspase-3 assay kit ((Sigma SCP0084)), anti-β-actin (A2547), anti-rabbit-secondary antibody (Sigma A0545), and anti-mouse-secondary antibody (Sigma A9044) were purchased from Sigma (St. Louis, MO, USA). Resveratrol was kindly given by Chongqing Kerui Nanhai Pharmaceutical Company and a 500 mM stock solution was made in DMSO (0.1% *v*/v final concentration) stored at − 80 °C. Antibody against phospho-LKB-1 (sc-271,934), Bcl-2 (sc-492), AMPK (sc-19,128), phospho-AMPK (sc-101,630), Bax (sc-6236), and Beclin-1 (sc-11,427) were purchased from Santa Cruz Biotechnology Inc. (Santa Cruz, CA, USA). Compound C (sc-200,689) and Z-DEVD-FMK (sc-311,558) were also purchased from Santa Cruz Biotechnology Inc. Antibody against cleaved caspase-3 was purchased from Cell Signaling Technology (Danvers, MA, USA). Antibodies against Bid (bs-1153R) were purchased from Beijing Biosynthesis Biotechnology Co., Ltd. (Beijing, China). RPMI 1640 medium was obtained from Gibco and fetal bovine serum was purchased from Shanghai Excell Biological Technology Co., Ltd. (Shanghai, China). Annexin V, propidium iodide (PI) and the caspase-3 inhibitor Ac-DEVD-CHO (C1206) were obtained from the Beyotime Institute of Biotechnology (Jiangsu, China).

### Cell lines and treatments

The human promyelocytic leukemia cell line HL-60 (ATCC® CCL240™) was purchased from the American Type Culture Collection (ATCC, USA). HL-60 cells were commonly cultured in RPMI 1640 medium supplemented with 10% (*v*/v) fetal bovine serum containing 100 U/ml penicillin and 100 U/ml streptomycin at 37 °C in a humidified atmosphere with 5% CO_2_. All experiments were performed only when the cells were growing during the exponential phase.

### Cell proliferation assay

7 × 10^3^ of exponentially growing cells were seeded in 96-well plates, and RSV of different concentration of 12.5–100 μM was added 24 h later. After incubation for 24 or 48 h, 20 μl of 1 mg/ml final concentration MTT(3-(4,5-dimethylthiazol-2-yl)- 2,5-diphenyltetrazolium bromide) was added, followed by a 4 h incubation at 37 °C, then 100 μl SDS-triplex liquid (10% SDS/5% isopropanol/12 mM HCl) was added to each well. The plate was then shaken for 10 min in the dark, then the absorbance value at 492 nm was read using a Micro-plate Auto-Reader (Labsystems). The percentage of cell viability was calculated as follows: cell viability (%) = (OD treatment/OD control) × 100% [10, 19].

### Flow cytometry analysis

An annexin V-PI staining kit (Santa Cruz) was used to analyze the apoptosis using flow cytometry according to the manufacturer’s manual and our protocol published before [9]. Briefly, cells were collected and resuspended in PBS after treatment, then annexin V-FITC and PI were added into the binding buffer. The log fluorescence values of annexin V-FITC (518 nm, FL1) and PI (620 nm, FL2) were shown on the X and Y axis, respectively.

### Caspase-3 activity

Caspase-3 activity was analyzed using a commercial caspase-3 assay kit according to the our previous work [20]. HL-60 cells were harvested and washed twice with ice-cold PBS after treatment. Cells were placed on ice for 15 min after being lysed by addition of 100 μl lysis buffer. Lysates were centrifuged at 15,000 g for 15 min followed by the protein concentrations assay using Bradford method.

### Western blot analysis

Cells were harvested, washed with cold PBS and the cells pellet was lysed using ice-cold RIPA (50 mM Tris–HCL pH 7.4, 150 mM NaCl, 1 mM EDTA, 1% Triton X-100, 1% sodium deoxycholate, 0.1% SDS, 1 mM PMSF, 5 mM aprotinin, leupeptin and pepstatin, 10 mM sodium orthovanadate and sodium fluoride) for 30 min. 50 μg protein was loaded and separated by SDS-PAGE on a 10 or 12% polyacrylamide gel. Proteins were transferred onto a nitrocellulose membrane and blocked with 5% non-fat milk in TBST buffer (50 mM Tris–HCl, pH 7.4, 0.15 mM NaCl, 0.1% Tween 20) for 30 min at room temperature. Blot membranes (Millipore, Bedford, MA, USA) were incubated (gently shaking) with 1st antibodies overnight at 4 °C. Blots were washed with TBST three times, 10 min each wash and then incubated with a 1:10,000 dilution of horseradish peroxidase-conjugated secondary antibody for 1 h at room temperature. Finally, the blot membranes were visualized with a SuperSignal West Dura detection kit (Pierce, Rockford, IL, USA) and exposed to medical Blue X-ray film after washed 3 times in TBST as we described previously [19].

### RNA extraction and PCR

Total RNA was extracted from HL-60 cells with Trizol reagent (Invitrogen) and reversely transcribed using a Primescript® RT reagent kit purchased from Takara (DRR037A) as described previouly [19]. PCR was performed using primers designed by Primer Premier 5.0 (Premier, Canada). The primers sequences were listed as follows: Beclin-1,5’-AGGTTGAGAAAGGCGAGACA-3′ and 5’-GCTTTTGTCCACTGCTCCTC-3′; the P62: 5’-AGCGTCAGGAAGGTGCCATT-3′ and 5’-TTCTCAAGCCCCATGTTGCAC-3′, and data were normalized using GAPDH transcript levels as internal control(GAPDH primers 5’-GGCCTCCAAGGAGTAAGACC-3′ and 5′- AGGGGAGATTCAGTGTGGTG-3′.) The amplified PCR products were separated by 2.0% agarose gel electrophoresis [19].

### Immunofluorescence staining and microscopy

It is referred as to our previously published work [9]. Briefly, Cells were collected and fixed with 4% formaldehyde at room temperature for 10 min. After rinsing with ice-cold phosphate-buffered saline (PBS) for three times, cells were permeabilized with 0.1% Triton X-100 for 10 min, and then blocked with 10% bovine serum albumin (BSA) for 1 h at room temperature. Cells were stained with a 1:500 dilution of corresponding primary antibodies overnight at 4 °C and incubated with CY3-conjugated anti-rabbit secondary antibody for 1 h at RT in the dark after washing with PBS for three times. DAPI was used to counterstain the nucleus and then mounted in glycerol followed by observing using a fluorescence microscope (Zeiss, Germany).

### Fluorescence intensity analysis

For quantitative analysis of fluorescence intensity, cells treated with various concentrations of RSV for the indicated time were stained with fluorescence dye. Subsequently, equal numbers of cells were transferred to black 96-well culture plates and then assayed with a Multiskan Spectrum (Molecular Devices, SpectraMax M2e 200–100) at a 507 nm excitation wavelength and 529 nm emission wavelength for Rho123. The relative fluorescence intensity of all treated group was calculated supposing the control group as 100%.

### siRNA transfection

For siRNA interference, cells were transfected using serum and antibiotics free culture medium containing Lipofectamine 2000 according to the manufacturer’s instructions and our protocol previously published [9]. Briefly, the target sequences were 5′-GGAGCCAUUUAUUGAAACUTT-3′ (sense) and 5′-AGUUUCAAUAAAUGGCUCCTT-3′ (antisense) for Beclin1, 5′-GUGACGAGGAAUUGACAAUTT-3′ (sense) and 5′-AUUGUCAAUUCCUCGUCACTT-3′ (antisense) for P62. 5′-GACGUUGGUAACUGACAAATT-3′ (sense), 5′-AGUUUCAAUAAAUGGCUCCTT-3′ (antisense) for ATG5, 5′-GCCCUCUACUGAUUGUUAATT-3′ (sense) and 5′- UUAACAAUCAGUAGAGGGCTT-3′ (antisense) for LC3. Cells were directly used for follow-up experiments 24 h later after transfection.

### Statistical analysis

Statistical analysis was performed using SPSS 15.0 software (SPSS Inc., Chicago, IL, USA). Data are expressed as means ± SD. Student’s unpaired t test was used to access differences between two values. A *p*-value of < 0.05 was considered to be statistically significant.

## Results

### RSV inhibits cell proliferation and induces cell death in HL-60 cells

**Fig. 1:**
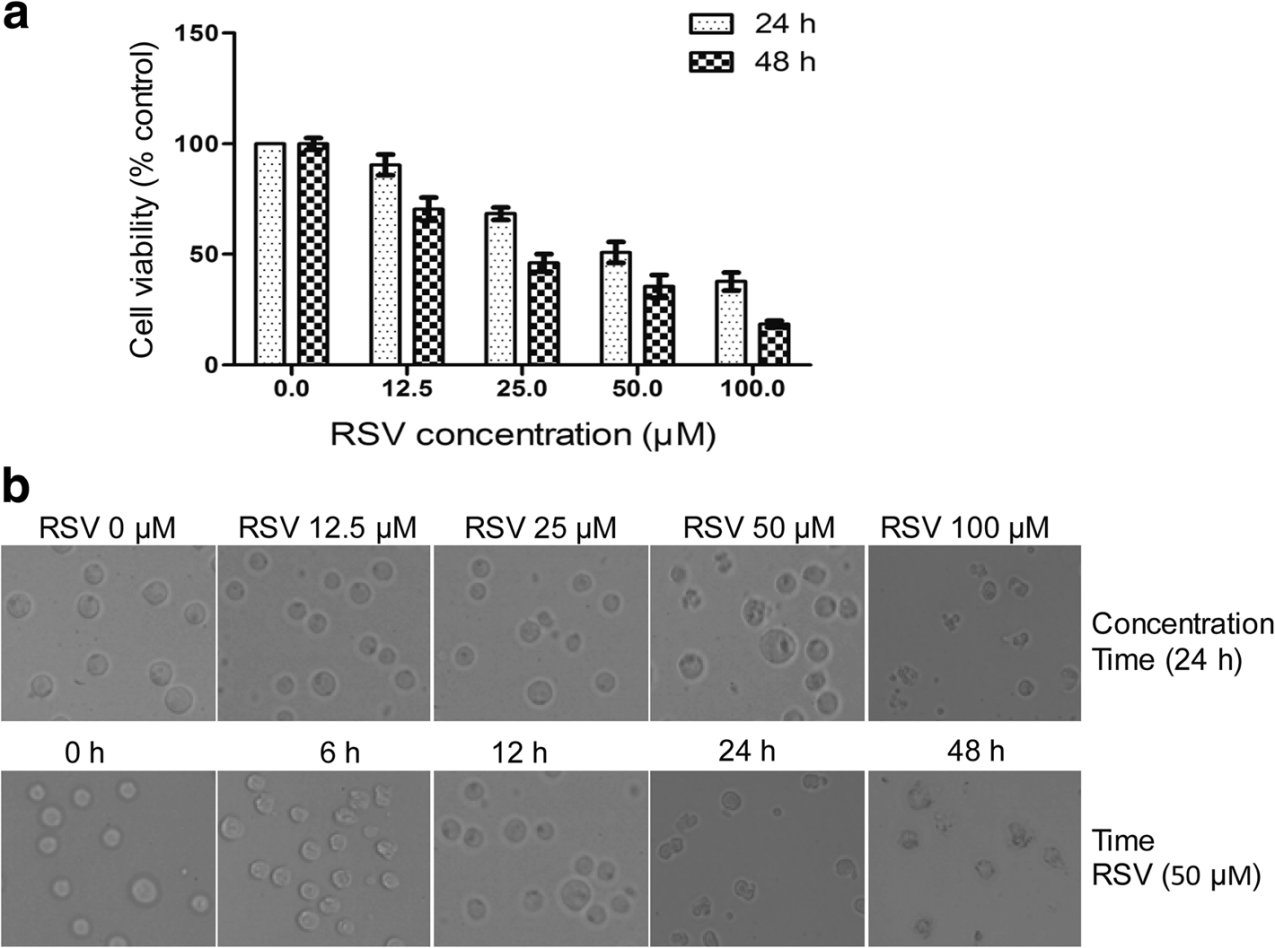
Determination of cytotoxicity and morphological changes of HL-60 cells after treatment with different doses of RSV or 50 μM RSV for different times. **a** HL-60 cells were treated with different concentrations of RSV for 24 and 48 h, then evaluated for cell viability by MTT assay. **b** HL60 cells were treated with different doses of RSV for different times as indicated, then changes in cell morphology were observed under an optical microscope. Data are presented as mean ± S.D. of three independent experiments in duplicates. *p < 0.05, vs. control

To investigate the action of RSV on the cell growth, HL-60 cells were incubated with different doses of RSV for 24 or 48 h, then cell viability was measured by MTT assay. As shown in Fig. 1a, RSV caused a dose- and time-dependent inhibition of cell growth. The IC50 of RSV on HL-60 cells was approximately 50 μM after 24 h treatment and nearly 30 μM after 48 h treatment. These results indicate that RSV blocks cell proliferation and induces cell death. Figure 1b shows that HL-60 cells, treated with different doses of RSV for various times, and then cell morphological changes were observed under an optical microscope, this data showed these characteristics of RSV-induced cells contain large fragments, cytoplasmic condensation, nuclear fragmentation and cell surface blebbing, indicative of apoptosis.

### RSV induces apoptosis in HL-60 cells

**Fig. 2:**
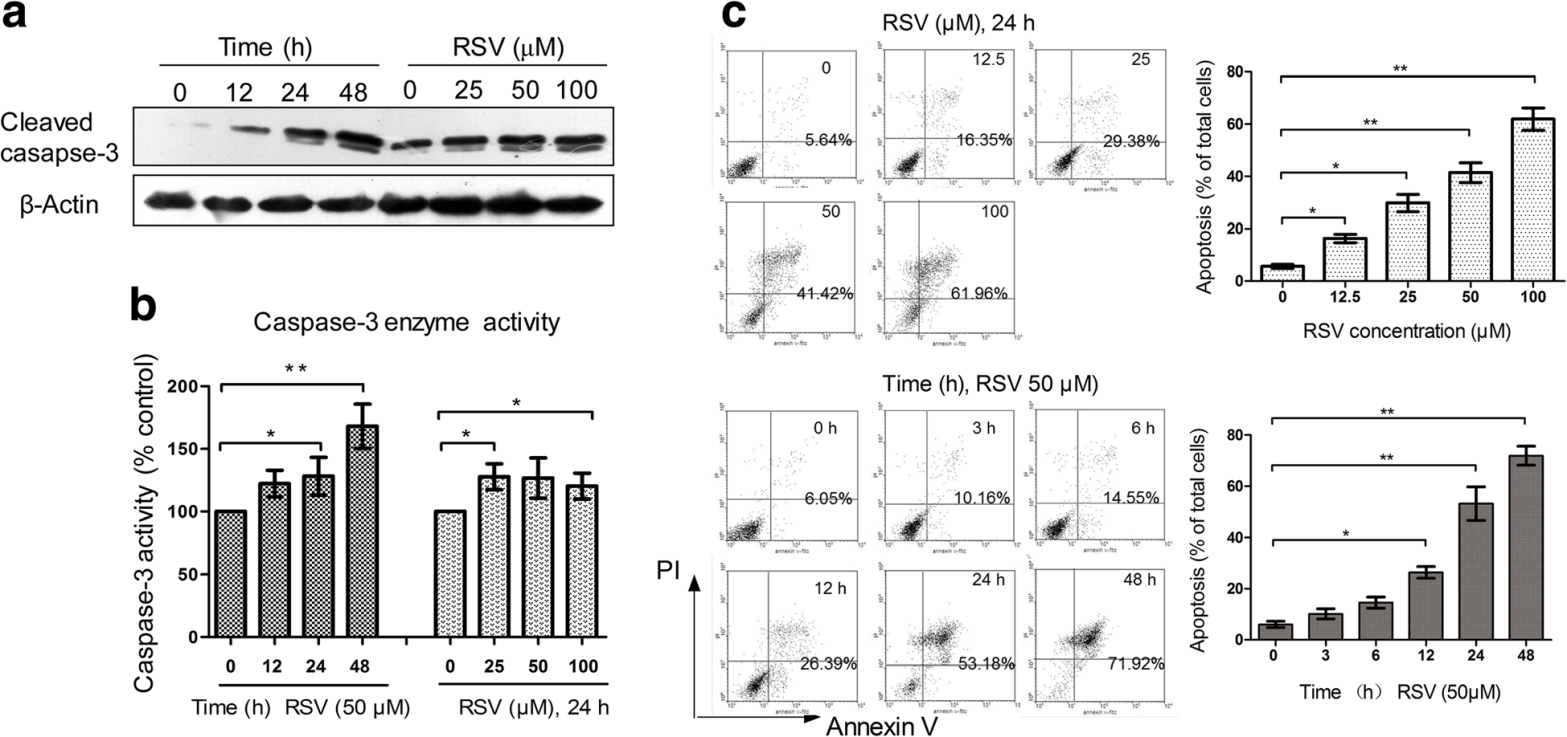
RSV induces HL-60 cell apoptosis in a dose- and time-dependent manner. **a** HL-60 cells were treated with different concentrations of RSV for 24 h, or treated with 50 μM RSV for different times, and western blot analysis was used for analyzing the expression of cleaved caspase-3. β-Actin was used as an internal control for equal amounts of protein applied. **b** HL-60 cells were treated with different concentrations of RSV for 24 h, or treated with 50 μM RSV for different times, then caspase-3 enzyme activity was analyzed using a commercial kit. **c** HL-60 cells were treated with different concentrations of RSV for 24 h, or treated with 50 μM RSV for different times, then cell apoptosis was analyzed by flow cytometry. The histogram represents quantification analysis based on three independent experiments. Columns indicate mean ± SD of three experiments, *p < 0.05 vs. respective control cells

Apoptosis is a normal physiological phenomenon in programmed cell death. In our experiment, we treated HL-60 cells with different doses of RSV for different times, then analyzed apoptosis-associated markers following RSV treatment. Western blot analysis revealed RSV induced caspase-3 cleavage in both a time- and dose- dependent manner (Fig. 2a). In monitoring caspase-3 enzymatic activity, the activity of caspase-3 also showed the same pattern as western blotting (Fig. 2b). To further confirm apoptosis, we next analyzed apoptotic cells by flow cytometry, following annexin V-PI double-staining, to more quantitatively detect the number of apoptotic cells. In agreement with the above results, with time and dose, the ratio of apoptotic cells increased (Fig. 2c). These results suggest that RSV induces apoptosis in a time- and dose-dependent manner in HL-60 cells.

### RSV induces both extrinsic and intrinsic apoptosis in HL-60 cells

**Fig. 3:**
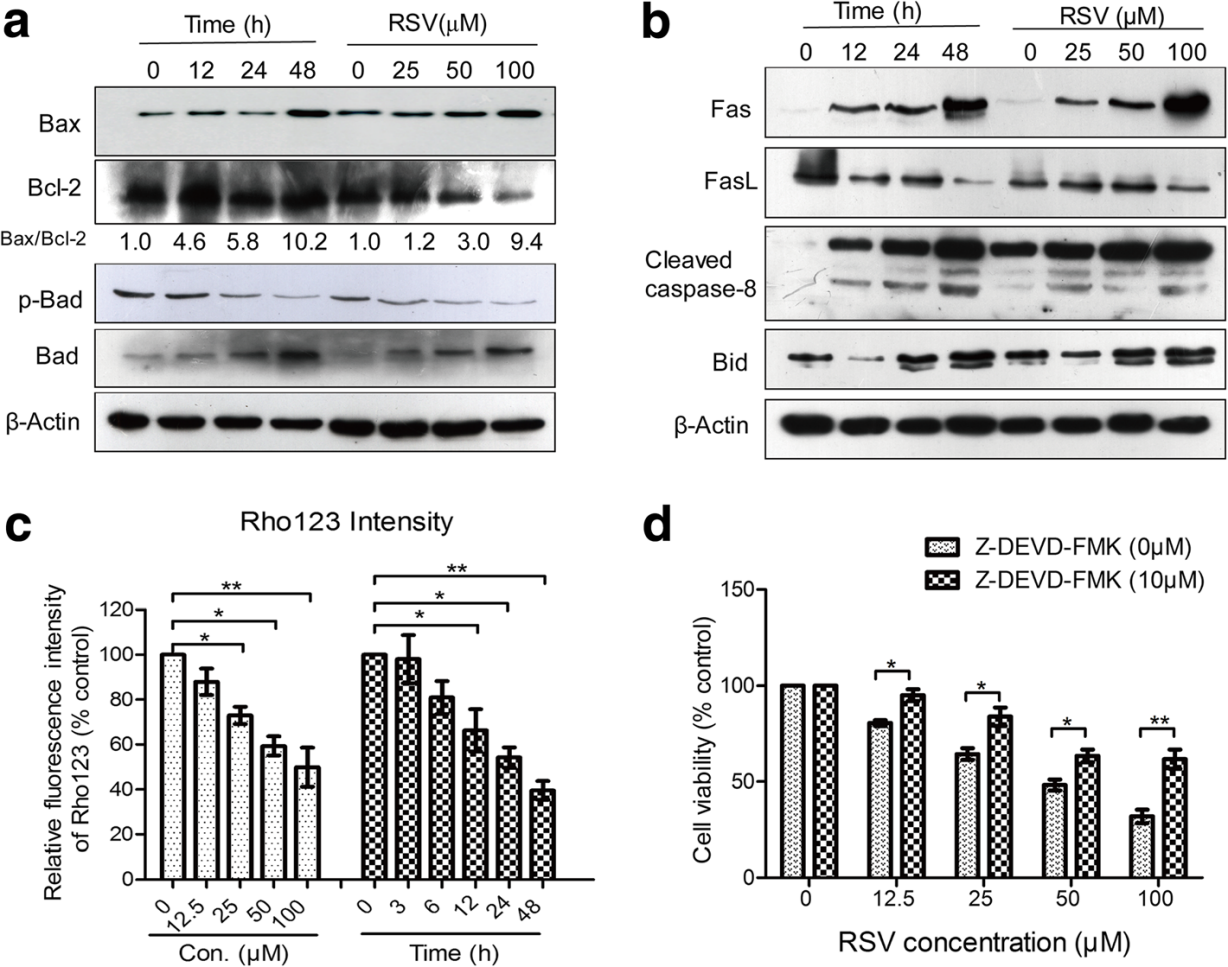
RSV induces HL-60 cell apoptosis through both the death receptor pathway and mitochondrial pathway. **a** HL-60 cells were treated with different concentrations of RSV for 24 h, or treated with 50 μM RSV for different times, then western blot analysis was used for analyzing the expression of proteins related to the intrinsic pathway of apoptosis by probing for Bax, Bcl-2, p-Bad and Bad. β-Actin was used as a loading control. **b** HL-60 cells were treated with different concentrations of RSV for 24 h, or treated with 50 μM RSV for different times and western blot analysis was used for analyzing the expression of extrinsic apoptosis pathway-related proteins by probing for Fas, Fas-L, Cleaved caspase-8, and Bid. β-Actin used as a loading control. **c** HL-60 cells were treated with 0–100 μM RSV for 24 h or 50 μM RSV for 0~ 48 h, then the mean fluorescence intensity of Rho123 was analyzed. **d** HL-60 cells were treated with different concentrations of RSV alone or combined with Z-DEVD-FMK (10 μM) for 24 h, then cell proliferation was analyzed by MTT assay. Data are presented as mean ± S.D. of three independent experiments in duplicates. *p < 0.05, vs. control

To illustrate the actual mechanism of RSV induced apoptosis, we analyzed both intrinsic and extrinsic apoptosis pathways. The intrinsic apoptosis pathway is also referred to as the mitochondrial apoptosis pathway. Extrinsic apoptosis refers to apoptosis initiated with Fas Ligands followed by caspase-8 cleavage to an activated form, which subsequently cleaves and activates caspase-3 or induces Bid cleavage, causing Bid to translocate to the mitochondria and induce apoptosis via the intrinsic pathway. We first examined the intrinsic pathway by detecting Bcl-2 family members (Fig. 3a). The ratio of Bax to Bcl-2 was increased in a time- and dose-dependent manner after RSV treatment. In addition, the expression of p-Bad was decreased, but total Bad was with an opposite trend (Fig. 3a). These results suggest that RSV induces apoptosis through the intrinsic mitochondria pathway in HL-60 cells.

Examination of the extrinsic apoptosis pathway, by western blot (Fig. 3b), revealed that the expression of Fas, and Fas-L were increased, and caspase-8 was cleaved in a time- and dose-dependent manner. Bid is a protein that is cleaved by caspase-8 and the cleavage fragment t-Bid then translocates to mitochondrial membrane to initiate the intrinsic pathway. Our results also show that Bid was cleaved, in accordance with tBid-mediated activation of the intrinsic pathway. To evaluate if apoptosis induced by RSV involves the mitochondria, we examined mitochondrial membrane potential (MMP) using Rho123 staining. The results showed that MMP decreased in a time- and dose-dependent manner with RSV treatment (Fig. 3c).

To further confirm that RSV induced apoptosis through both extrinsic and intrinsic apoptosis pathways, we then used the caspase-3 inhibitor, Z-DEVD-FMK to co-treat the cells with RSV. The combined treatment with RSV and caspase inhibitors showed that RSV-mediated reduction in cell viability is reversed by inhibitors of caspase-3 (Fig. 3d). These data further confirm our hypothesis that both extrinsic and intrinsic pathways are involved in RSV-induced apoptosis.

### RSV induces autophagy in HL-60 cells

**Fig. 4:**
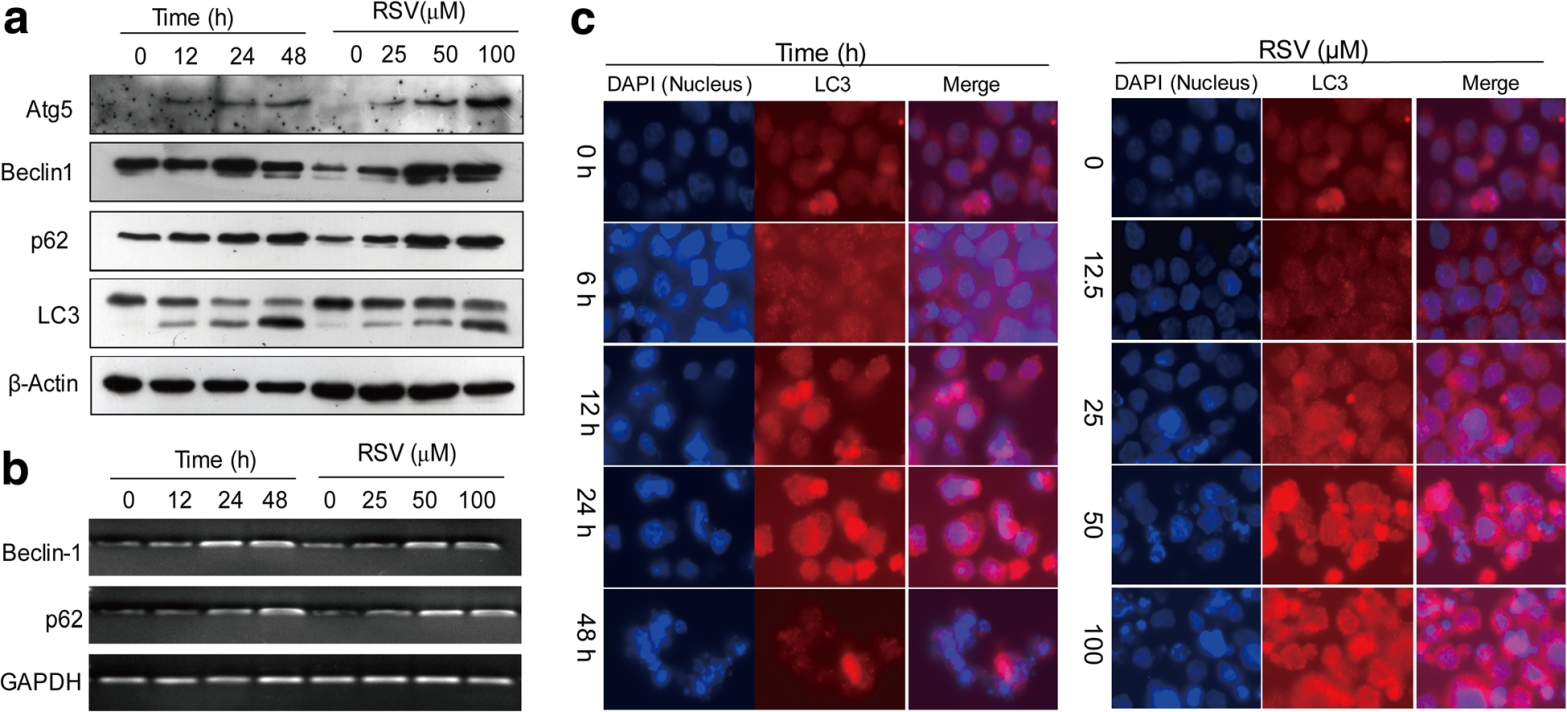
RSV induces time- and dose-dependent autophagy in HL-60 cells. **a** HL-60 cells were treated with different concentrations of RSV for 24 h, or treated with 50 μM RSV for different times, and western blot analysis was used for analyzing the expression of autophagy-related proteins by using the specific antibodies as indicated in figure. β-Actin was used as a loading control. **b** HL-60 cells were treated with different concentrations of RSV for 24 h, or treated with 50 μM RSV for different times, then semi-quantitative PCR was used for detecting the expression of autophagy-related genes Beclin-1 and P62. GAPDH was used as a control. **c** HL60 cells were treated with different doses of RSV for different times as indicated, then immunofluorescence was used for analyzing the expression of the autophagy marker, LC3. Data are presented as mean ± S.D. of three independent experiments in duplicates. *p < 0.05, vs. control

Autophagy is a process that may function either as an attempt for cell survival under certain conditions, or as a second type of programmed cell death [21]. We and others have shown that RSV can induce autophagy in different cancer cell lines [19, 22]. Therefore, we checked whether RSV has the ability to induce autophagy in HL-60 cells. We treated HL-60 cells with RSV for various time periods and at various concentrations, then examined cells for molecular markers of autophagy by western blot analysis (Fig. 4a). Consistent with autophagy, the autophagy-specific marker, LC3, was converted from type I to type II, following RSV treatment, in a time- and dose-dependent manner. We also found that other autophagy-related genes, such as Atg5 and Beclin-1, were increased under the action of RSV in a time- and dose-dependent manner. P62, an autophagy-related protein that recruits other proteins for degradation during autophagy, was also increased in a time- and dose-dependent manner.

Analysis of these autophagy-related genes by RT-PCR was consistent with the western blot results (Fig. 4b). Detection of the autophagy marker LC3 by immunostaining showed increased expression of LC3II speckles detected in cells treated with RSV in a time- and dose-dependent manner (Fig. 4c). These results indicate the induction of autophagy in HL-60 cells exposed to RSV.

### Activation of the LKB1-AMPK pathway and inhibition of PI3K-AKT signaling contribute to mTOR inhibition by RSV-induced autophagy

**Fig. 5:**
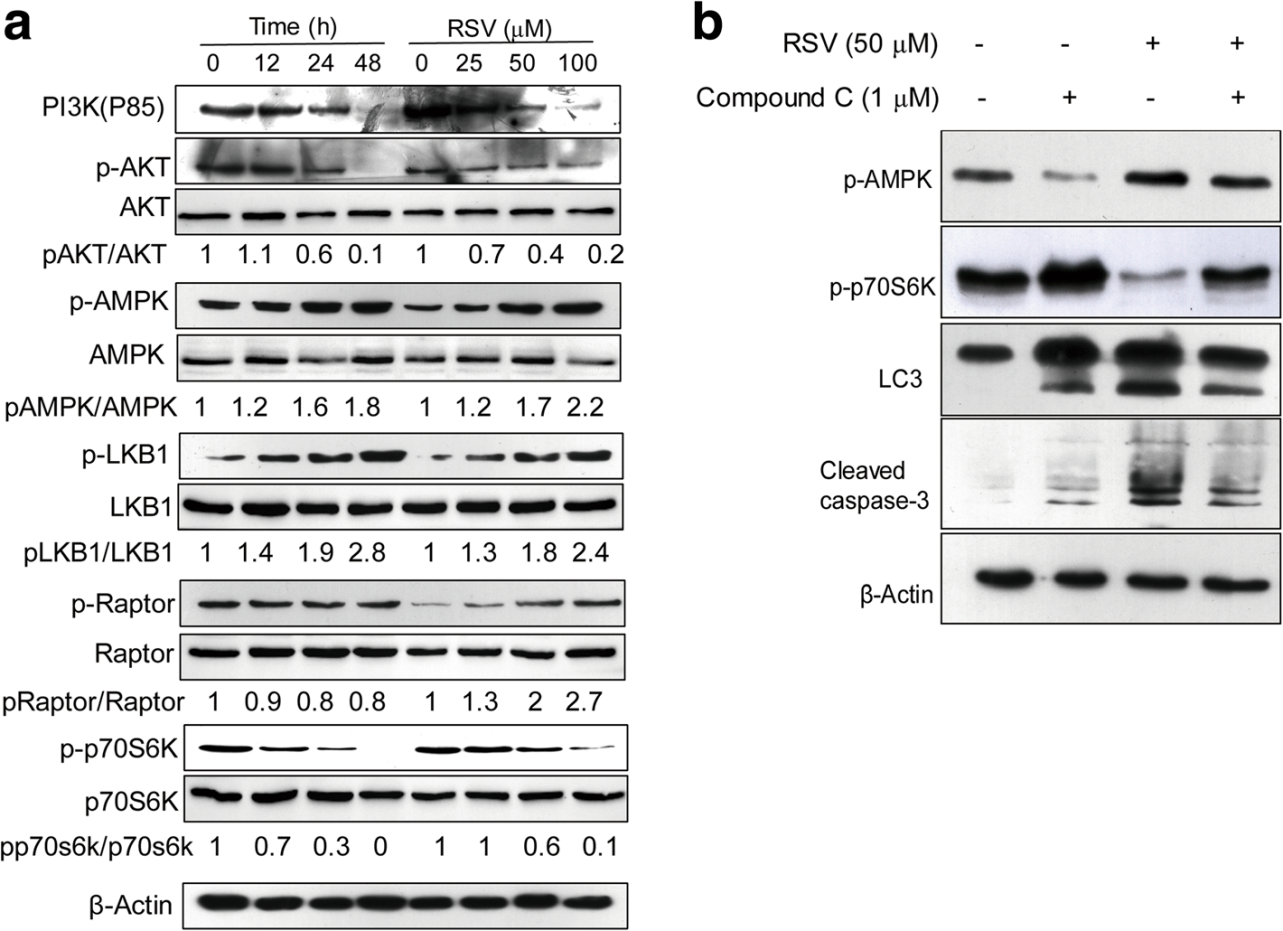
Autophagy induced by RSV in HL-60 cells depends on the PI3K-AKT and LKB1-AMPK-mTOR pathways. **a** HL-60 cells were treated with different concentrations of RSV for 24 h, or treated with 50 μM RSV for different times, then PI3K(P85), p-AKT/AKT, p-LKB1/LKB1, p-Raptor/Raptor and p-p70S6K/p70S6K were analyzed by western-blot. β-Actin was used as an internal control to show equal amounts of protein were applied. **b** HL-60 cells were incubated with 50 μM RSV combined with compound C (1 mM) for 24 h, then P-AMPK, P-P70S6K, LC3 and cleaved caspase3 were analyzed by western blot analysis with the corresponding antibodies. β-Actin was used as the loading control. Data are presented as mean ± S.D. of three independent experiments in duplicates. *p < 0.05, vs. control

mTOR is a serine/threonine protein kinase involved in many cell processes, such as cell proliferation and protein synthesis. We and others have shown that mTOR is a negative regulator of autophagy, and that inhibition of mTOR could activate autophagy. In this study, inhibition of the mTOR pathway occurred following treatment with RSV. Phospho-p70S6K, which is directly phosphorylated by mTOR, was also detected by western blot. Results showed that p-p70S6K was decreased by RSV treatment in a time- and dose-dependent manner (Fig. 5a). We also see from Fig. 5a that the PI3K-AKT pathway was inhibited after RSV treatment, when probed with antibodies against PI3K (p85) and p-AKT, but that the LKB1-AMPK pathway was activated by RSV, as shown by the increased pattern of p-AMPK and p-LKB1. Raptor has been identified as a direct substrate of AMPK. Phosphorylation of raptor is essential for inhibition of the raptor-containing mTOR. We found that p-Raptor was increased following treatment with RSV (Fig. 5a), consistent with RSV inducing p-AMPK activation. From the above data, we conclude that both the PI3K-AKT and LKB1-AMPK pathways are activated in the treatment of RSV and their activation contributes to autophagy.

To further confirm that autophagy induced by RSV involves the AMPK-mTOR pathway, we then combined compound C, an AMPK inhibitor, with RSV, to treat HL-60 cells. Cells were pre-treated with compound C for 1 h followed by RSV treatment, the AMPK-mTOR pathway was clearly inhibited compared with cells treated with RSV only, as judged by western blot analysis for p-AMPK and p-p70S6K. Conversion of the autophagy-related marker LC3 from type I to type II also decreased (Fig. 5b). From these data, we conclude that the activation of AMPK-mTOR pathway is involved in RSV-induced autophagy, since it can be inhibited by compound C. In conclusion, our data reveals that RSV-induced activation of the LKB1-AMPK-mTOR pathway and inhibition of the PI3K-AKT pathway are involved in activation of autophagy.

### RSV-induced autophagy enhances apoptosis and triggers cell death in HL-60 cells

**Fig. 6:**
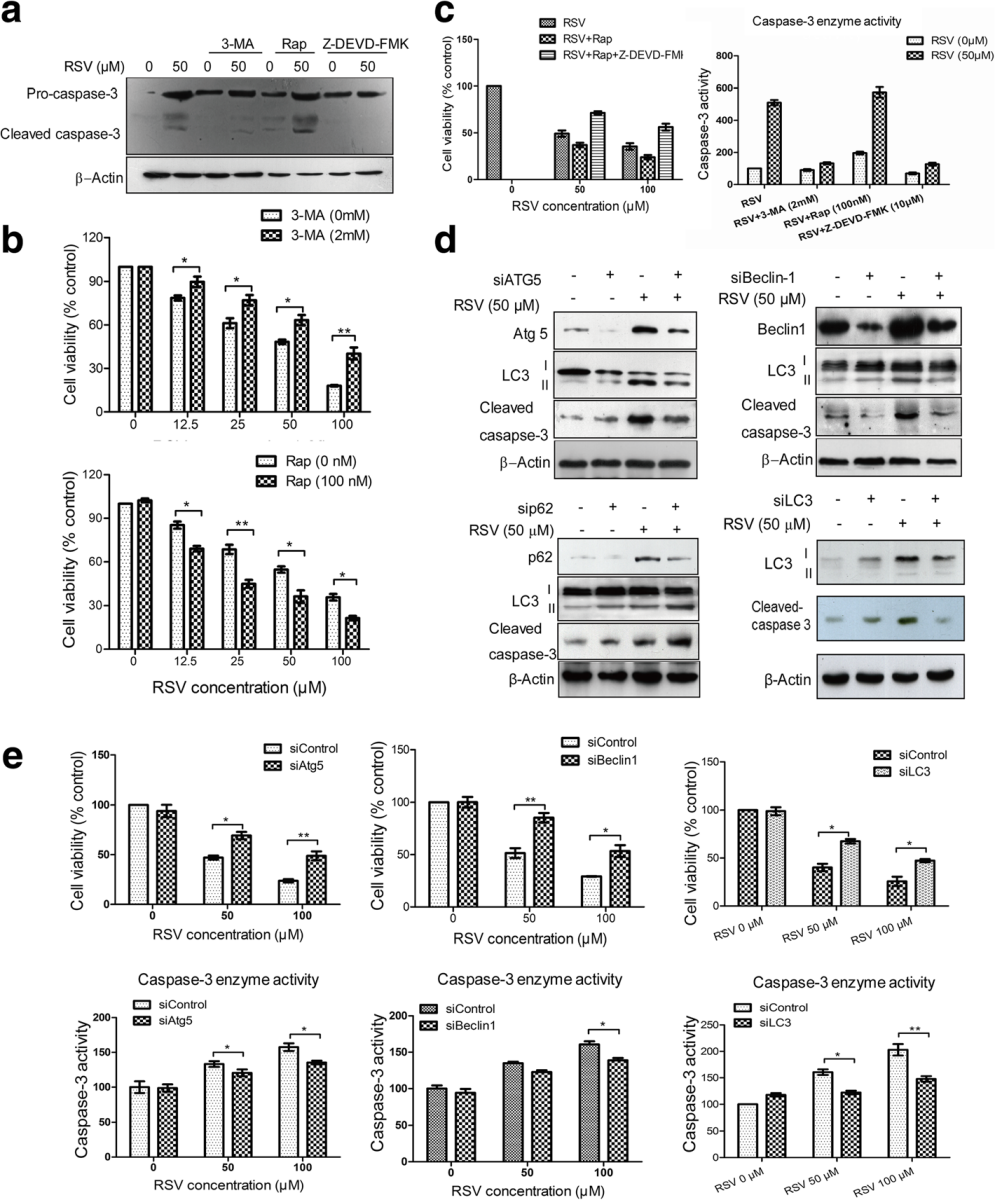
RSV induces HL-60 cells to undergo autophagic cell death through enhancing apoptosis. **a** HL-60 cells were treated with the indicated drugs for 24 h, and western blot analysis was used for analyzing casapse-3. β-Actin was used as the loading control. **b** HL-60 cells were treated with RSV alone or combined with 3-MA (2 mM) or rapamycin (100 nM) for 24 h, then cell viability was detected by MTT assay. **c** HL60 cells were treated with RSV combined with rapamycin, 3-MA or the caspase inhibitor Z-DEVD-FMK for 24 h. Caspase-3 enzymatic activity was then analyzed using a commercial kit, and cell viability was detected by MTT assay. **d** HL-60 cells were transfected with siAtg5, siBeclin-1, siP62 or siLC3, then cells were treated with different doses of RSV for 24 h. Western blot analysis was then used for analyzing the expression of autophagy- and apoptosis-related proteins. β-actin was used as the loading control. **e** HL-60 cells were transfected with siRNA for Beclin-1, Atg5, LC3. Cells were then incubated with different doses of RSV for 24 h, then caspase-3 enzyme activity was quantified and cell viability was detected by MTT assay. Columns indicate the mean ± SD of three experiments, * *p* < 0.05 vs. respective control cells

Our data shows that RSV induces both autophagy and apoptosis in HL-60 cells. To further delineate the relationship between autophagy and apoptosis, we first examined the effects of the autophagy inhibitor 3-MA and inducer rapamycin in apoptosis. From Fig. 6a, showing cleaved caspase-3, we also can conclude from these data that 3-MA inhibits apoptosis, but rapamycin has a small effect on RSV-mediated apoptosis. We next examined cell viability following treatment with either the autophagy inhibitor or inducer. 3-MA increased cell viability after RSV treatment combined with 3-MA, but rapamycin decreased cell survival (Fig. 6b). Then we detected cell viability and caspase-3 enzyme activity under conditions of co-treatment with RSV, rapamycin, and a caspase-3 inhibitor. When autophagy was stimulated by combined treatment with RSV and rapamycin, inhibition of caspase-3 resulted in an increase in cell viability and a decrease in caspase-3 enzyme activity (Fig. 6c). All the above data suggest that autophagy induced by RSV may enhance apoptosis and induce cell death.

To further confirm that autophagy enhances apoptosis, we then knocked down autophagy-related genes by siRNA. When cells were transfected with siAtg5, siBeclin1 and siLC3, autophagy marker LC3-II was decreased compared with the control siRNA-transfected group (Fig. 6d). Cleaved caspase-3 was also decreased following inhibition of autophagy by addition of Beclin1, ATG5 and LC3 siRNA. However, on the contrary, when cells were transfected with siP62, cleaved caspase-3 was increased (Fig. 6d). Analysis of caspase-3 enzyme activity further confirmed our western blot data. We next detected cell viability after transfection with the above autophagy-related genes (Fig. 6e). From these data, we conclude that RSV-induced autophagy enhances apoptosis and triggers cell death in HL-60 cells.

## Discussion

Previous studies have shown that RSV can inhibit the proliferation of human promyelocytic leukemia cells and induce apoptosis [10, 23, 24]. Studies on the mechanisms involved in the RSV induced-autophagy have also attracted much attention [11, 25]. RSV has also been proved to have an antiproliferative effect in many tumors [20, 26, 27]. Although many studies have also shown that RSV acts against cancer by inducing apoptosis, the underlying mechanisms involved in RSV-induced cancer cell death is not clear. In the present study, we provide a better understanding on RSV-induced cell death and interaction of apoptosis and autophagy in this model. We further show that RSV-induced apoptosis is reduced after the inhibition of autophagy by pharmaceutical and genetic inhibitors of autophagy. RSV induces autophagy through the PI3K-AKT and LKB1-AMPK-mTOR pathways. However, apoptosis, in our model, is activated through both the intrinsic and extrinsic pathways.

The mTOR pathway activation is reported to be involved in the autophagy inhibition in various types of cancers [28]. Rapamycin, a widely used mTOR inhibitor, has been demonstrated to be applied in cancer therapy by suppressing the proliferation of cancer cells through inducinig autophagy [29]. Here, we first confirm that autophagy induced by RSV depends on mTOR, and that RSV inhibits the activity of mTOR. Furthermore, we examined the LKB1-AMPK-mTOR and PI3K-AKT pathways, up-stream activators of mTOR, and show that RSV-mediated inhibition of mTOR is dependent on both the LKB1-AMPK and PI3K-AKT pathways.

Loss of mitochondrial membrane potential is reported to be one of important cell apoptotic markers [30] and allows the release of pro-apoptotic molecules (eg. cytochrome C), from mitochondria to the cytosol [9, 31]. In our study, we confirm that RSV can induce apoptosis by decreasing mitochondrial membrane potential and triggering caspase-3 activation. It has been previously reported that RSV could induce apoptosis through both intrinsic and extrinsic pathways [32]. We examined related markers of these two separate pathways. We first showed that the Fas and Fas-L expression level is enhanced after RSV treatment and that caspase-8 cleavage induces truncation of Bid, which then translocates to the mitochondria and induces apoptosis. Thus, our data shows that the two pathways are both activated, consistent with previous reports. Our data also shows that autophagy and apoptosis are enhanced by RSV, similar to our prior publication on A549 cells [19], as well as work from other laboratories [33, 34], leading us to study whether autophagy and apoptosis occur at the same time, or whether any correlation exists between these two processes.

In the present study, we found that 3-MA potently attenuates the cytotoxicity of RSV on HL-60 cells. It demonstrates that both apoptosis and autophagy contribute to cell death, as is consistent with results from blocking autophagosome formation with Atg5 and Beclin1 gene knockdown. Therefore, blocking autophagy induced by RSV reduces cell death. Interestingly, knockdown of P62 also enhances caspase-3 dependent apoptosis, possibly because the decreased level of p62 prevents the degradation of LC3, enabling cell death to occur by autophagy.

This study systematically examines the role of apoptotic and autophagy in RSV induced cell death. Although previous study have been reported to find RSV can induce cell death through apoptosis [23, 35], It is the first time for us to find that apoptosis and autophagy involved in the RSV induced cell death simultaneously in HL-60 cells, as indicated by increased markers for autophagy or apoptosis. On this basis, we found that the cell death rate is proportional to the concentration of resveratrol, and increases with the extension of time; whereas after inhibition of autophagy, RSV-mediated cell death is dramatically lower. Conversely, treatment with autophagy inducers increased cell death. So in this study, we demonstrate that RSV can induce cell death in HL-60 cells through both apoptosis and autophagy. Our results also suggest that autophagy may be a process to enhance apoptosis, as suggested by others [22, 36, 37]. However, to our knowledge, our study is the first to comprehensively illustrate the crosstalk between autophagy and apoptosis, and autophagy-dependent apoptosis under treatment with RSV.

## Conclusions

This study presents a novel mechanism involving concurrent autophagy and apoptosis in RSV-induced cancer cell death model. RSV activates both Fas ligand-mediated and mitochondrial apoptosis in HL-60 cells by increasing Bax expression, cytochrome C release into the cytosol, and subsequent activation of caspase-3. We examine the mechanisms of RSV-induced cell death in HL-60 cells and demonstrate that RSV-induced autophagy is a necessary process to apoptosis. To sum up, our research proves that cell autophagy plays an important role and it is the inevitable process of the cell apoptosis in the model of resveratrol induced cell death of HL-60 cells.

## Additional files

Additional file 1: **Figure S1.** Caspase 3 inhibitor Z-DEVD-FMK decreased RSV induces HL-60 cell apoptosis. HL-60 cells were treated with different concentrations of RSV, or cotreated with 10 μM Z-DEVD-FMK for 24 h, then cell apoptosis was analyzed by flow cytometry. The histogram represents quantification analysis based on three independent experiments. Columns indicate mean ± SD of three experiments, *p < 0.05 vs. respective control cells. (TIF 305 kb)

Additional file 2: **Figure S2.** Western blot analysis of autophagy and apoptosis signal proteins induced by RSV in the control HL-60 cells**.** (A) HL-60 cells were treated with 0 μM of RSV for 0,24 and 48 h as control cells, then pro-caspase3, caspase3, ATG5, Beclin1, p62 and LC3, (B) p-AKT/AKT, p-AMPK/AMPK, and p-p70S6K/p70S6K were analyzed by western-blot .β-Actin was used as an internal control to show equal amounts of protein were applied. Data are presented as mean ± S.D. of three independent experiments in duplicates. (TIF 197 kb)

## Abbreviations

3-MA: 3-methyladenine
DAPI: 4,6-diamidino-2-phenylindole
DMSO: Dimethyl sulfoxide
LC3: Light chain 3
MMP: Mitochondrial membranes potential
RSV: Resveratrol, 3, 4′, 5-trihydroxy- trans-stilbene
siRNA: Small interfering RNA
SQSTM1: Sequestosome1
Z-DEVD-FMK: Z-Asp(OMe)-Glu(OMe)-Val-Asp(OMe)-FMK
Z-IETD-FMK: Z-Ile-Glu (OMe)-Thr-Asp(OMe)-FMK
