# Supplementary figures and images for: Resveratrol induces autophagy-dependent apoptosis in HL-60 cells
Source: BMC Cancer. 2018 May 22;18:581. doi: 10.1186/s12885-018-4504-5 (PMC5964919; doi:10.1186/s12885-018-4504-5)

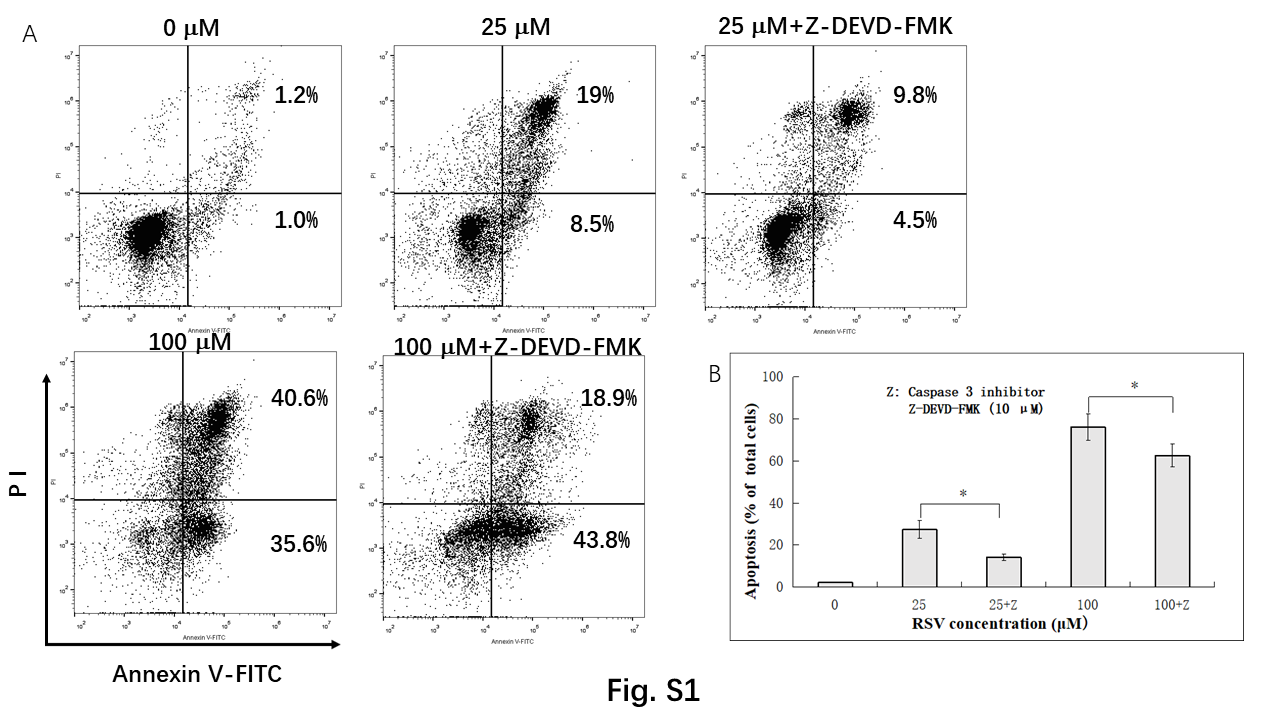

Supplement: Supplementary file 1 — Figure S1. Caspase 3 inhibitor Z-DEVD-FMK decreased RSV induces HL-60 cell apoptosis. HL-60 cells were treated with different concentrations of RSV, or cotreated with 10 μM Z-DEVD-FMK for 24 h, then cell apoptosis was analyzed by flow cytometry. The histogram represents quantification analysis based on three independent experiments. Columns indicate mean ± SD of three experiments, *p < 0.05 vs. respective control cells. (TIF 305 kb) [file 12885_2018_4504_MOESM1_ESM.tif]

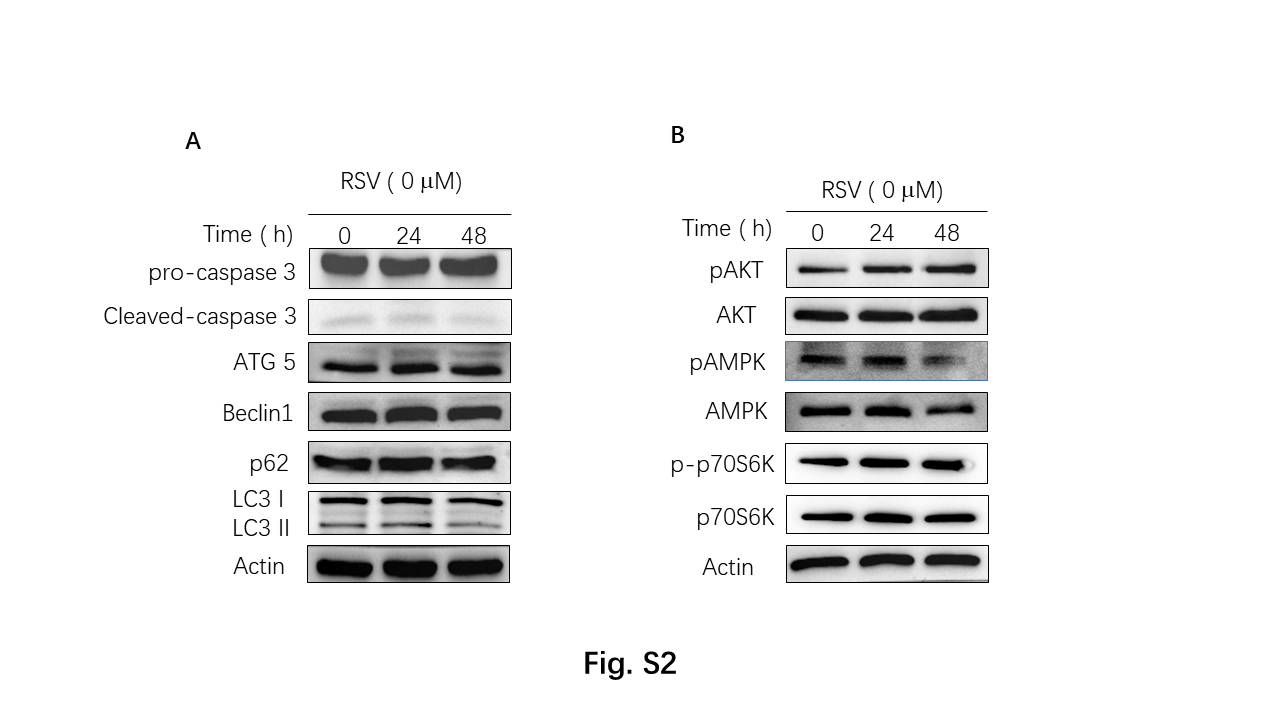

Supplement: Supplementary file 2 — Figure S2. Western blot analysis of autophagy and apoptosis signal proteins induced by RSV in the control HL-60 cells. (A) HL-60 cells were treated with 0 μM of RSV for 0,24 and 48 h as control cells, then pro-caspase3, caspase3, ATG5, Beclin1, p62 and LC3, (B) p-AKT/AKT, p-AMPK/AMPK, and p-p70S6K/p70S6K were analyzed by western-blot .β-Actin was used as an internal control to show equal amounts of protein were applied. Data are presented as mean ± S.D. of three independent experiments in duplicates. (TIF 197 kb) [file 12885_2018_4504_MOESM2_ESM.tif]
